# Does ‘Data fudging’ explain the autocratic advantage? Evidence from the gap between Official Covid-19 mortality and excess mortality

**DOI:** 10.1016/j.ssmph.2022.101247

**Published:** 2022-09-30

**Authors:** Eric Neumayer, Thomas Plümper

**Affiliations:** a Department of Geography & Environment, London School of Economics and Political Science (LSE), London, UK; b Department of Socioeconomics, Vienna University of Economics and Business, Vienna, Austria

## Abstract

Governments can underreport Covid-19 mortality to make their performance appear more successful than it is. Autocracies are more likely to ‘fudge’ these data since many autocratic regimes restrict media freedom and thus can prevent domestic media from reporting evidence of undercounting deaths. Autocracies also enjoy greater leverage over reporting health authorities to either fudge data or adopt restrictive definitions of what constitutes Covid-19 mortality. Controlling for other factors that explain official Covid-19 mortality, excess mortality and the difference between the two, our results suggest that any apparent ‘autocratic advantage’ in fighting the pandemic is likely to only exist in official Covid-19 mortality. Analyzing the gap between excess mortality and official Covid-19 mortality we find that autocracies on average have a larger gap between official Covid-19 mortality data and excess mortality data, which points towards ‘autocratic data fudging’ of their official Covid-19 mortality statistics.

## Highlights

• Autocracies have greater incentives and opportunity to underreport Covid-19 mortality.
• Excess mortality provides a check on the reliability of official Covid-19 mortality.
• Autocracies seemingly experience lower official Covid-19 mortality rates.
• Autocracies exhibit a larger gap between excess and reported Covid-19 mortality.
• More autocratic countries are more likely to ‘fudge’ their Covid-19 mortality data.

## 1 Introduction

The evidence supporting the existence of an ‘autocratic advantage’ (Cepaluni et al., 2021; Chen et al., 2021; Karabulut et al., 2021; Sorci et al., 2020) in the fight against the Covid-19 pandemic is weak. Recent research has demonstrated that the positive statistical association between democratic regime type and official Covid-19 mortality rates disappears when researchers either control for data transparency (Annaka, 2021) or for a battery of political and institutional differences between autocracies and democracies. In fact, Cassan and Steenvort (2021: 13) suggest: “Under the assumption that our extensive set of controls captures the determinants of COVID 19 mortality rates, (…) autocracies may be manipulating their reported COVID 19 death rate.”

Demonstrating that conditional on a particular set of control variables, regime type no longer has a predicted positive effect on official Covid-19 mortality rates is instructive but does not directly substantiate the claim that autocracies manipulate Covid-19 death statistics. Research more directly focusing on data fudging has shown that Covid-19 mortality data published by autocratic governments are more likely to violate Benford's law and related regularities in data that occur naturally (Adiguzel et al., 2020; Kapoor et al., 2020). Benford's law predicts a distribution of the first digit of data when the underlying population grows approximately exponentially and when the range of data points is large enough – a factor of 10,000 between the minimum and the maximum is generally accepted as necessary for identifying data fudging (Fewster, 2009; Miller, 2015). In most countries the range of reported Covid-19 mortality is smaller, often much smaller. For example, Belarus never reported more than 11 deaths per day before August 18, 2021. As the conditions for Benford's law are not given other than in very large countries, one should be cautious to interpret a violation of Benford's law as sufficient evidence for data fudging (Campolieti, 2021; Koch & Okamura, 2020; Sambridge & Jackson, 2020; Silva & Figueiredo Filho, 2021).

We adopt a different research design that is also directly aimed at providing evidence for data fudging. Specifically, we analyze the gap between excess mortality on the one hand and official Covid-19 mortality on the other hand. Such a focus on excess mortality has been pioneered by studies that aim at demonstrating the ‘true cost’ of the pandemic (Karlinsky & Kobak, 2021; Sanmarchi et al., 2021). Some authors promote the analysis of excess mortality estimates as “gold standard” (Beaney et al., 2020; Jain et al., 2021) for the evaluation of the political response to Covid-19. We show the challenge but also great promise in adopting this approach. The main challenge stems from the fact that a positive gap does not necessarily imply that countries underreport Covid-19 deaths (Böttcher et al., 2021; Staadegaard et al., 2021). Official records count only deaths that are officially attributed as having been caused, in full or as a contributory factor, by a Sars-CoV-2 infection. By contrast, excess mortality estimates quantify all the direct but also the *indirect* mortality effects of the pandemic and of containment policies implemented to keep the pandemic under control (Karlinsky & Kobak, 2021). Still, this approach is promising if, as we will argue, the systematic factors driving a wedge between excess mortality and official Covid-19 mortality can be sufficiently controlled for. We show that autocracies only have a seeming advantage over democracies in official Covid-19 mortality. By contrast, analyzing the gap between excess mortality and official Covid-19 mortality we find that autocracies on average exhibit a larger gap than democracies which suggests that they fudge their official Covid-19 mortality data.

## 2 The politics of data fudging

Cases of data fudging of official records by governments and public administrations have rarely been detected, prosecuted and condemned. Governments seem to have little to fear when they fudge data and no government has as yet been taken to court for underreporting Covid-19 mortality data. Data fudging is, it appears, a cavalier offence.

### 2.1 Why fudge?

The publication of data in general and of Covid-19 mortality data in particular communicates information from which the population derives inferences about the government's performance. Governments fudge data if they wish to appear more successful than they actually are. The intriguing question is why don't all governments fudge data? Put differently: what is the political constellation that prevents some governments from fudging?

As noted above, there is some tentative evidence that autocratic governments underreport Covid-19 mortality data while democratic governments do not – or autocrats underreport more and more often. However, this appears puzzling: why would governments that do not face competitive elections choose to cheat while governments that face the real prospect of being voted out of office and may therefore have stronger incentives to appear successful abstain from data fabrication and manipulation? Hollyer et al. (2015) argue that data transparency stabilizes the government in democracies but triggers protest and unrest in autocracies. Autocratic governments that face potential opposition therefore have incentives to fudge, while in democracies the opposite holds: transparent and on the whole honest communication of data and other information to voters stabilizes the government.

We suggest a different causal mechanism. Building on a traditional political economy approach we assume that data fudging involves political trade-offs.

On the one hand, underreporting Covid-19 mortality data generates the impression of successful containment policies. Success is important for incumbents in democratic regimes that need to win elections. However, success is also important for autocratic governments that do not allow free and open electoral competition, because, as Hollyer at al. (2015: 764) contend, “autocratic governments, despite their seemingly unconstrained authority, live in the shadow of mass political unrest. At any given moment, the public may reject the existing political order and – through action (strikes/protests) in the streets – impose substantial costs upon their leaders, sometimes even ousting the leadership or upending the regime.” Adiguzel et al. (2020) similarly argue that governments in autocratic countries have more to fear from the economic and social problems prompted by the pandemic, which can cause significant political disturbance. Autocratic leaders also face the threat of a coup d’état. If they do not manage to provide sufficient rents to the political, economic and military elites of their country, they face severe opposition and the threat of being replaced by alternative leaders. Hence, all governments have an incentive to misrepresent relevant data to let their containment policies appear more successful, not just democracies that face competitive elections. Since individuals are much more afraid of the mortality risk posed by the virus than of the risk of becoming infected as such, there is a particularly strong incentive for governments to misrepresent Covid-19 mortality data.

On the other hand, however, misrepresenting mortality data gives a false impression of safety and suggests that Covid-19 is not more dangerous than the flu, a notion that not only leads to a decline in risk-averse behaviors but also incentivizes governments to implement less stringent containment policies (Becher et al., 2021). In turn, the transmission rate of the virus increases. Hence, if governments and health administrations underreport mortality data to appear more successful, they potentially facilitate the spread of the virus, increase infections, and ultimately increase actual Covid-19 mortality.

Autocratic governments, we submit, are more likely than their democratic counterparts to underreport Covid-19 mortality for predominantly two reasons: first, autocrats do not depend as much as democratic governments on protecting the health of the general population. Many autocratic governments therefore invest less in the public health infrastructure (Blum et al., 2021) and they also care less about the actual as opposed to official Covid-19 mortality. And, second, democracies usually have health institutions that collect and report data independently of the government and they have free media that are likely to report irregularities in Covid-19 data (Solis & Waggoner, 2021). By contrast, autocratic governments have more leverage over reporting health administrations and often restrict media freedom. Thus, even if it is plausible that democratic governments face similar incentives as autocracies to fudge Covid-19 mortality data, they have less opportunity to do so and face a higher risk of being caught if they did engage in data fudging.

### 2.2 Why focus on mortality data?

If governments have incentives to misrepresent data related to Covid-19, why focus on mortality data? There are two principal reasons for this focus. Firstly, there are reasons why governments intent on fudging Covid-19 data should focus on mortality data. Fudging official Covid-19 mortality data downwards is comparably easy. Health administrations do not even have to fabricate data. A very restrictive definition of what counts as Covid-19 mortality suffices to “cause” low Covid-19 mortality. Also, public and scientific perception has focused on Covid-19 deaths in the evaluation of the success of containment policies (Fotiou & Lagerborg, 2021; Checo et al., 2021; Chen et al., 2021; Sachs, 2021; House of Commons, 2021). And secondly, even if those governments that manipulate Covid-19 mortality data may also manipulate other data relevant to the pandemic, excess mortality figures allow researchers to detect potential data fudging in mortality data, whereas no comparable benchmark data exist for, say, under-reporting in infection rates.

It is true that a country set on fudging its official Covid-19 mortality data may also fudge its total mortality data or, as Turkey has done, simply stop reporting those. As Ariel Karlinsky, one of the lead researchers tracking excess mortality, explains: “Turkey is a prime example of a place where they have the numbers but they are not releasing them because they do not want to explain the discrepancies.” The Turkish government tried to avoid the detection of potentially serious excess mortality that would have spoiled the narrative of a safe destination for tourists. However, we know of no other country that has followed this approach or where there are indications of fudging total mortality data. This may well be because governments learned too late into the pandemic that total mortality figures can be used to estimate excess mortality which can be employed to check on the accuracy of officially reported Covid-19 mortality.

## 3 How reliable are Official Covid-19 mortality records, excess mortality estimates and the mortality gap between the two?

Evidence that demonstrates a substantively important difference between official Covid-19 mortality records and excess mortality continues to grow. By now, such a Covid mortality gap has been shown to exist in many countries (IMHE, 2021; The Economist, 2021; WHO, 2022). This research aims at, as Katie Pierce from Johns Hopkins university suggests, uncovering the “true impact of Covid-19”.^1^ This claim is not unproblematic as we will show in this section, in which we discuss how reliable Covid-19 mortality figures are, how reliable excess mortality estimates are and what systematic factors other than a desire to underreport can drive a gap between the two.

### 3.1 How reliable are Official Covid-19 mortality data?

The reliability of official Covid-19 mortality statistics depends on the definition of Covid-19 mortality, on the ability of doctors to identify the main cause of death and the ability of public health authorities to correctly collect, aggregate and report data. Much of the measurement uncertainty with official Covid-19 data boils down to what evidence is required to classify a death as ‘caused by Covid-19’. An uncontroversial definition of Covid-19 mortality does not exist (Karanikolos & McKee, 2020). Identification of Covid-19 mortality is impeded by the fact that co-morbidities increase the probability of dying from an infection with Sars-CoV-2. As always, causal inference is hampered because the counterfactual is unobservable – one simply does not know if and when a Covid-19 patient would have died in the absence of the infection.

Three factors have a major impact on who is counted in official Covid-19 statistics. First, some countries only accept a death as Covid-related if the deceased had tested positive for Sars-CoV-2 before they died. This leads to undercounting in countries that test little (The Economist, 2021). Other countries only count deaths if the deceased have been hospitalized because of Covid before they died. The exclusion of those who die of Covid but not in a hospital leads to undercounting. Some countries appear to adopt this definition as Riffe and Acosta (2021: 390d) point out: “Some populations only report deaths occurring in hospitals, neglecting a potentially sizeable proportion of deaths occurring in institutional settings and at home.” Second, some countries only count people who have died within the first three or four weeks after being tested positive. If a patient dies with multiple organ failures long after she got infected it is assumed that she died of multiple organ failures and not of Covid – regardless of whether these failures have been caused by the virus or not. This results in undercounting. On the other hand, some of the infected people who die within a certain period of time may have died in any case even in the absence of infection, typically due to severe co-morbidities. This results in overcounting.

Third, some countries rely on cause-of-death assessments either solely or in addition to other criteria of what counts as Covid-19 mortality. The latter can help reduce the overcounting mentioned above. As Riffe and Acosta (2021: 390d) explain: “Most populations currently report all deaths to confirmed SARS-CoV-2 infections as COVID-19 deaths for this database, but the underlying cause of death eventually reported on the death certificate may differ in patients with severe comorbidities.” The reliability of death certificates is a direct function of the availability of coroners and their competence in making such judgements. People who have died for unknown reasons may be classified as Covid-19 deaths as this is easier than finding the true cause.

### 3.2 How reliable are excess mortality estimates?

Excess mortality data “sidestep” (Leon et al., 2020) the conceptual difficulties in attributing mortality to Covid-19. The term excess mortality describes the difference between the observed number of deaths in a given period and the typically to be expected number of deaths in the same period based on the number of deaths that occurred during that period over the course of usually the last four or five years. The historical average can, however, sometimes be corrected for unusual events that happened during this historical time, which means that the historical average is estimated rather than simply taken from death tables. Thus, if a country has a positive excess mortality figure in January 2020 more people died in that month than had died on average during January in the years 2016–2019.

In order to assess the reliability of excess mortality estimates, one must distinguish their reliability in general from their specific reliability as a measure of Covid-19 mortality. The accuracy of excess mortality data suffers from delays in reporting deaths. Not all countries have the capacity to register and report all deaths on a frequent and reliable basis. As a consequence, excess mortality estimates only exist for a subset of countries: “Excess mortality can only be calculated on the basis of accurate, high-frequency data on mortality from previous years. But few countries have statistical agencies with the capacity and infrastructure to report the number of people that died in a given month, week or even day-to-day. For most low- and middle-income countries, such data is not available for previous years.”^2^

More problematic is the specific reliability of excess mortality as a superior measure of Covid-19 mortality. Excess mortality does not only capture Covid-19 fatalities, but all changes in mortality that have been recorded relative to the reference period. It therefore captures all positive and indeed negative deviations from the historical average whether or not they are directly related to the virus or only indirectly caused by the myriad consequences of the pandemic. Clearly, this Covid-19 pandemic has severely negatively affected the health care system of all countries that suffered from high incidence rates and it has deterred some people from seeking health care, thus increasing the morbidity of other life-threatening diseases.

At the same time, the pandemic and the containment measures may have increased the propensity for other serious diseases and conditions, including depressions (Ettman et al., 2020). Research has found a small increase of suicides in some countries due to containment measures, which increases excess mortality. Likewise, there is evidence for an increase in drug abuse (Taylor et al., 2021). Yet, the pandemic also reduces some risks (Kamdi & Deogade, 2020). During the early days of the pandemic, the number of traffic accidents had declined. Most remarkably, however, social distancing measures had reduced the mortality from influenza to virtually nil.^3^ In ‘normal years’, those to which the mortality figures during the course of the pandemic are compared to, the excess mortality observed during winter months (relative to other months) is typically assumed to be driven by influenza fatalities. During the winter seasons of 2020/21 and 2021/22 influenza was essentially absent, which has, all other things equal, lowered excess mortality during these months.

### 3.3 How reliable is the gap between excess mortality and official Covid-19 mortality?

As we have seen, both official statistics of Covid-19 deaths and excess mortality data are problematic. However, this does not imply that little can be learned from analyzing the deviation between both types of information for the purpose of detecting potential data fudging in Covid-19 mortality statistics. The usefulness of the difference between the two measures depends on our ability to control for the systematic ways in which excess mortality captures the indirect impact of the pandemic beyond direct Covid-19 mortality. Table 1 provides an overview of potential sources of error, their effect on both official Covid-19 mortality statistics and excess mortality and how this impacts the reliability of the gap between the two as a measure of Covid-19 mortality underreporting. Table 1 also suggests options for controlling for these potential sources of error.

**Table 1:** A systematic categorization of sources of error and their expected effects.

source of error	official C-19 mortality	excess mortality	reliability of gap between excess and official as measure of underreporting	control option
severity of the pandemic	+	++	upward bias	incidence rate age structure of population vaccination rate
health system overburdened	+	++	upward bias	GDP p.c. incidence rate vaccination rate
pandemic induced change in other causes of death, principally influenza	0	–	downward bias	stringency of containment policies
				hemisphere-specific period fixed effects
low state capacity	–	–	unclear bias	government effectiveness

We have identified four sources of potentially systematic measurement error, which can bias upwards or downwards the gap between excess mortality and official Covid-19 mortality records as a reliable measure of Covid-19 mortality underreporting. At the same time, one cannot rule out that these sources of error are correlated with political incentives to let the mortality data look better than they are, in which case it is important to control for them.

The first source of error stems from the severity of the pandemic in the country, which pushes up both official Covid-19 mortality and excess mortality, but we expect the influence on excess mortality to be larger as indirect collateral mortality damage from the pandemic also becomes exacerbated. The more severe the pandemic the more people will stay away from hospital for non-Covid related medical conditions and even avoid routine care by general medical practitioners for fear of becoming infected. We control for the severity of the pandemic not only by including the incidence rate but also by two important factors that influence mortality per infected person, namely the median age of the population and the vaccination rate.

The second source of bias is the quality of the health system before the pandemic and the effect the pandemic has had on the effectiveness of the health system. A health system that is ineffective will result, all other things equal, in higher Covid-19 mortality. Poor quality of the health system pre-pandemic and an overburdened health system during the pandemic will push upwards Covid-19 mortality but also indirect excess mortality not directly related to Covid-19 mortality. Ineffective health systems will fail more easily to cope with medical problems people have independently of Covid-19 and particularly so when it is under stress from a large number of Covid-19 patients with operations and routine care either cancelled or delayed. Unfortunately, reliable measures of the pre-pandemic *quality* of the health system do not exist for a large number of countries. One could use data on health spending per capita but this does not necessarily reflect quality. Instead, we suggest to use gross domestic product per capita since richer countries typically enjoy better quality health systems either via government provision or via the market. To account for the stress the pandemic itself has exerted on the health system one would ideally include a variable counting the share of free hospital and intensive unit care beds but since these variables do not systematically and reliably exist cross-nationally, the already introduced incidence and vaccination rates can function as a proxy for pressure on health systems since higher incidence rates translate into higher hospitalization rates whereas vaccines have strongly reduced hospitalization and thus pressure on the health system.

The third and related potential for bias emanates from the influence of the pandemic on mortality from other causes of death. Multiple adverse consequences exist beyond the increase in mortality resulting from other diseases when and where the health system is at the brink of collapse from Covid-19 patients or because patients shy away from seeking health care that we already discussed above. For example, the pandemic impacts on suicides and drug-related fatalities because of social isolation. Much more substantively importantly, social distancing measures had a very strong dampening effect on the transmission of the influenza virus and other infectious diseases. The reduction in mortality from other infectious diseases does not affect official Covid-19 mortality records but means that, all other things equal, excess mortality is lower than in ‘normal’ years such that the gap between excess and official Covid-19 mortality is biased downwards as an indicator of underreporting Covid-19 mortality. In addition to the incidence rate and vaccination rate as proxies for the stress Covid-19 has brought onto the health system, one can control for the expected systematic consequences of the policies, rules and regulations aimed at containing the pandemic by including a measure of the stringency of these policies, rules and regulations and one can also include hemisphere-specific period fixed effects to control for significant reduction in mortality from influenza and other infectious diseases during the winter period.

A fourth potential source of error stems from a low state capacity. Countries with poor public administration of the health sector have few laboratories able to conduct PCR tests and will therefore often fail to identify Sars-CoV-2 and register too few cumulative infections. If this underreporting of cases coincides with a definition of Covid-19 mortality that requires a positive test for Sars-CoV-2 before death, the underreporting of infections will ultimately lead to an underreporting of Covid-19 deaths. Countries with low state capacity will also have problems with establishing the true cause of death. Of course, low state capacity may not only downward bias official Covid-19 mortality data but also excess mortality estimates such that the overall effect on the gap between the two is unclear. There are numerous proxies one could use. We will employ the World Bank's indicator of a government's effectiveness, which relies on expert assessments of the capacity of government to provide public services, the quality of the bureaucracy, the competence of civil servants, the independence of the civil service from political pressures, and the credibility of the government's commitment to policies.

## 4 The correlates of Official Covid-19 mortality, excess mortality and the gap between the two

Controlling for systematic factors that impact official Covid-19 mortality, excess mortality and the gap between the two, we can estimate whether democracies fare systematically better or worse than autocracies on each of these. Before controlling for these systematic factors, we first of all look at the extent to which official records and excess mortality figures differ from each other without taking these factors into account and how this difference or gap is associated with democracy.

We source excess mortality from the World Health Organization (WHO)^4^ and official Covid-19 mortality data from ourworldindata.org. The WHO provides two fundamentally different types of estimates of excess mortality. The more reliable ones are labelled “reported” by the WHO because they are based on reported total mortality figures in the relevant country months. This gives us a sample of 93 countries in the estimations reported below. Recall that excess mortality data depend on the capacity to register deaths on a frequent and reliable basis and so poor countries, which on average also tend to be more autocratic than rich countries, are under-represented in this sample. In addition, the WHO also provides another type called “predicted” excess mortality data for other countries or, at times, for specific months in countries that in other periods report total mortality. These predictions are based on total mortality data derived from a statistical model using country-specific variables (see WHO, 2022 for details). Taking “reported” and “predicted” types of excess mortality data together gives a sample of 158 countries in total in our estimations. Countries are listed in appendix 1.

We base our preliminary analysis on the smaller sample based on the more reliable “reported” total mortality figures. Likewise, our interpretation of substantive effect sizes will be based on this sample but we report multivariate estimation results further below for both this and the full sample that includes excess mortality estimates based on predicted total mortality.

The temporal coverage is March 2020 extending to December 2021. Our measure of political regime type is based on the well-known liberal democracy score from the Varieties of Democracy project but further below we also describe results from robustness tests using two other measures from the Polity project and from Freedom House.^5^

We start our analysis with a brief description of how the average reported Covid-19 death rate, the excess mortality rate and the gap between excess and reported Covid-19 mortality varies by 0.1 steps of the liberal democracy score that theoretically runs from 0 to 1 (note though that no country scores above 0.9) for the restricted sample consisting of observations in which excess mortality estimates are based on reported total mortality data.

**Fig. 1:**
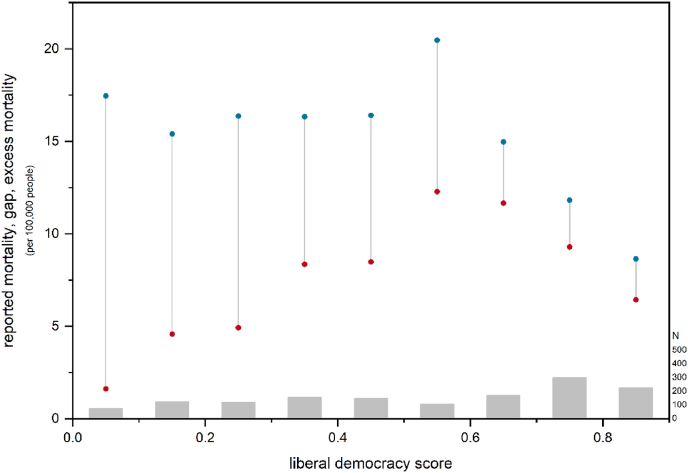
Official Covid-19 Mortality, Excess Mortality and the Gap by Liberal Democracy Category Note: red dot indicates reported Covid-19 mortality, blue dot excess mortality, the line between the dots indicates the gap; the bar charts indicate the frequency of observations in each category. . (For interpretation of the references to colour in this figure legend, the reader is referred to the Web version of this article.)

Fig. 1 reveals the existence of two patterns: official Covid-19 mortality rates are on average higher in more democratic countries, though the trend levels out at around a liberal democracy score of 0.6 – countries such as Slovenia, South Africa and Tunisia. Excess mortality figures do not show this trend and both official Covid-19 mortality rates and excess mortality rates are much lower in countries with the highest liberal democracy score – countries such as those in Western Europe but also Costa Rica – than in countries closer to the middle of the distribution of liberal democracy scores while excess mortality in countries with the highest liberal democracy score is also much lower than in those with a low score. The most striking finding from Fig. 1 is that the gap between excess mortality and reported Covid-19 mortality is much higher in autocratic countries than in democracies with the gap falling nearly linearly going from the most autocratic to the most democratic countries.

While Fig. 1 provides some tentative evidence for data fudging among at least some autocracies, simple comparisons between official mortality figures and excess mortality estimates are potentially misleading as we have discussed in the previous section. We therefore now control for the factors that we have identified as affecting, though differentially, both official Covid-19 and excess mortality and potentially undermining the reliability of the gap between official Covid-19 and excess mortality as a measure of underreported Covid-19 mortality. The explanatory variables consist of those listed in Table 1 and described in the previous section. See appendix 2 for sources of data, the time period they refer to and descriptive variable statistics. We lag the average monthly stringency of containment policies by 1 month as it takes time for policy changes to prompt behavioral changes resulting in changes to mortality. We estimate the models with ordinary least squares (OLS) with standard errors clustered on countries. The estimating equation, not displaying hemisphere-specific period fixed effects that are included in all regressions, is as follows:

DV_it_ = β_1_(liberal democracy)_it_ + β_2_(incidence rate)_it_ + β_3_(median age)_it_ + β_4_(% pop. fully vacc.)_it_ + β_5_(p.c. income (ln))_it_ + β_6_(stringency index)_it-1_ + β_7_(gov. effectiveness)_i_ + ε_it_

Where i refers to country, t refers to year month (note that some variables only vary from year to year as explained in appendix 2 and that government effectiveness has no time variation), ε_it_ is the error term and DV stands for the three dependent variables: official Covid-19 mortality, excess mortality and the gap between the two.

Table 2 presents our estimations results. We have two sets of estimations – one for the smaller sample of country months with excess mortality estimates based on the more reliable “reported” total mortality data only and another one for all country months including those in which total mortality is based on predictions – for, respectively, official Covid-19 mortality, excess mortality and the gap between excess mortality and official Covid-19 mortality as the dependent variables.

**Table 2:** Estimation results.

	official covid mortality rates, per 100k		excess mortality rates, per 100k		mortality gap	
sample:	reported total mortality only	all countries	reported total mortality only	all countries	reported total mortality only	all countries
model:	m1	m2	m3	m4	m5	m6
liberal democracy	4.472*	3.609**	−10.66*	−5.258	−15.13***	−8.867***
	(2.436)	(1.407)	(5.466)	(3.426)	(4.697)	(2.899)
incidence rate, per million people	0.000831***	0.000902***	0.00150***	0.00154***	0.000667***	0.000639***
	(9.74e-05)	(8.77e-05)	(0.000181)	(0.000166)	(0.000122)	(0.000109)
median age	0.369***	0.319***	0.676***	0.599***	0.307**	0.279***
	(0.0769)	(0.0608)	(0.168)	(0.128)	(0.138)	(0.0994)
% population fully vaccinated	−0.176***	−0.106***	−0.309***	−0.182***	−0.133***	−0.0760***
	(0.0343)	(0.0169)	(0.0637)	(0.0294)	(0.0389)	(0.0181)
per capita income (ln)	0.450	−0.297	−0.108	−0.955	−0.558	−0.658
	(0.573)	(0.365)	(1.695)	(0.851)	(1.531)	(0.648)
stringency index (t-1)	0.0939***	0.0706***	0.0336	0.0312	−0.0603*	−0.0395**
	(0.0219)	(0.0140)	(0.0434)	(0.0260)	(0.0332)	(0.0199)
government effectiveness	−3.810***	−2.213***	−5.821***	−3.658***	−2.011	−1.444*
	(0.790)	(0.522)	(1.718)	(1.093)	(1.410)	(0.795)
adj. R2	0.348	0.303	0.301	0.307	0.177	0.228
countries	93	158	93	158	93	158
N	1,725	2,974	1,725	2,974	1,725	2,974

Notes: all regressions contain hemisphere-specific period fixed effects. Standard errors clustered on countries in parentheses. *, **, *** statistically significant at 10, 5 and 1 percent, respectively.

Empirical results are largely as expected. We find a positive and statistically significant association between liberal democracy and official Covid-19 mortality – the finding that triggered the debate on the autocratic advantage. However, the perceived autocratic advantage does not only disappear when we analyze excess mortality, there is some evidence for an autocratic disadvantage. The statistical association between the liberal democracy score and excess mortality is negative, albeit only marginally statistically significantly so and only for the sample only based on reported total mortality. More importantly, liberal democracy is negatively and statistically significantly associated with the gap between reported Covid-19 mortality rates and excess mortality in both samples, a finding that indicates that democracies on average have lower mortality gaps compared to autocracies, which are therefore more likely to underreport Covid-19 mortality. On average, the most autocratic countries underreport Covid-19 mortality by 12.7 deaths per 100,000 people per month compared to the most democratic countries like those in Western Europe. For a hypothetical country of assumed population size of 50 million people this would translate into a higher gap between excess mortality and official Covid-19 mortality of 57,150 people over the sample period if this hypothetical country were as autocratic as possible in our sample as opposed to as democratic as possible.

**Fig. 2:**
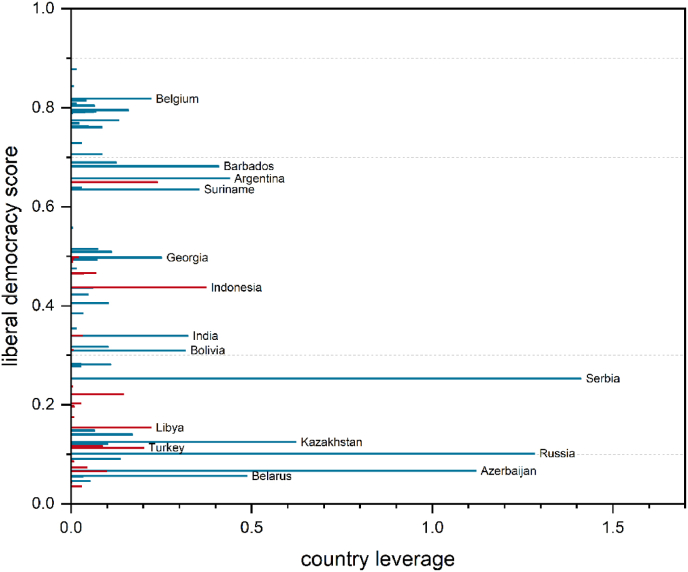
Country Leverage on the Effect of Liberal Democracy on the Difference between Official Covid 19 Mortality and Excess Mortality Note: Blue: excess mortality estimates based on reported total mortality; red: excess mortality estimates based on predicted total mortality.. (For interpretation of the references to colour in this figure legend, the reader is referred to the Web version of this article.)

Mean effects are not necessarily good indicators for the magnitude of data fudging because not all autocracies underreport Covid-19 mortality and those that do underreport have little to fudge in between major waves of Covid-19 when both incidence and Covid-19 mortality rates are low. Average effects also do not tell us anything about which are the most likely autocratic data fudgers. Admittedly, we cannot ‘identify’ any data fudging countries in the sense of identifying countries definitely guilty of data fudging. However, we can provide some further tentative evidence on which countries are likely to be among the data fudgers. To do so, we have estimated the statistical leverage of each country on the estimated coefficient of the liberal democracy score in model 6 with all countries included. We define leverage as the absolute difference in the coefficient of liberal democracy in the full sample minus the coefficient in the sample from which a country has been removed. Recall that the liberal democracy score has a negative effect on the gap between excess mortality and reported mortality figures: more liberal countries on average have a smaller mortality gap. This negative slope becomes smaller – that is, the coefficient becomes larger – if an autocratic fudger is removed from the sample. In Fig. 2 we display the leverage that the removal of a country exerts (note that we only display those countries whose removal renders the effect of the liberal democracy score less negative). As Fig. 1 suggests, Azerbaijan, Belarus, Kazakhstan, Russia and Serbia are all examples of countries with a low or very low liberal democracy score that our analysis would suggest as fudging their data.

Note that in Fig. 2 we indicate whether observations for a country refer to excess mortality estimates based on reported (blue) or predicted (red) total mortality data. The WHO's methodology is likely to underestimate the estimated excess mortality figures for countries that underreport official Covid-19 mortality data and do not report total mortality figures and for which the WHO therefore predicts total mortality because the WHO's prediction model includes the official Covid-19 mortality as one of the predictors (WHO, 2022, p. 6). Thus, if countries underreport Covid-19 mortality, the predicted excess mortality figures and ultimately our calculated gap is underestimated. This may partly explain why countries for which the WHO provides ‘predicted’ excess mortality figures are underrepresented in our estimate of potential fudgers and why the estimates in Table 2 for the full model differ from the estimates of the restricted but more reliable model that only covers observations in which countries report total mortality data. Turkey is a case in point. As we have explained above, the country stopped reporting total mortality data and is likely to have underreported its official Covid-19 mortality data to appear as a safe place for tourists. Our leverage calculation suggests that Turkey is likely to fudge, but Turkey's leverage is not very high. One explanation is that the WHO's predictions of total mortality underestimate the true level of excess mortality in Turkey. If this is true, then the true leverage score of Turkey is also higher than what we report here.

We have subjected our findings from Table 2 to a number of robustness tests, which account for the fact that any model specification is subject to uncertainty (Neumayer & Plümper, 2017). Due to space constraints, we only describe rather than fully report results from these tests but all results are included in the replication dataset and do-file. The liberal democracy score from the Varieties of Democracy project has arguably become the dominant measure of democracy in political science. If we replace this measure with the political rights measure from Freedom House or the democracy measure from the Economist Intelligence Unit then our results are very similar.^6^ The same holds for using the polity2 measure from Center for Systemic Peace except that whilst the negative coefficient of democracy in the regressions on the mortality gap remains statistically significant, albeit only marginally so at the 10 percent level, in the more reliable restricted sample of countries that report total mortality data it is no longer statistically significant in the full sample that includes also those countries with estimated rather than reported total mortality.^7^

One can question whether the effect of variables in the sample is approximately linear as assumed in Table 2. If we allow second- and third-order polynomials of explanatory variables then the goodness of model fit increases slightly but results are fully robust. Lastly, our unit of analysis in the estimations is a country month which help accounting for other time-varying factors impacting official Covid-19 mortality, excess mortality and the mortality gap. Our results are fully robust if we temporally aggregate to the yearly level instead.

## 5 Conclusion

Governments have incentives to underreport Covid-19 mortality and, not surprisingly, some governments seem to give in to this temptation. Accounting for factors that influence Covid-19 mortality, excess mortality and the gap between excess mortality and official Covid-19 mortality records, we found that the positive and statistically significant correlation between liberal democracy and official Covid-19 mortality not only disappears but is reversed: liberal democracies exhibit lower excess mortality and a lower gap between excess mortality and official Covid-19 mortality.

The apparent ‘autocratic advantage’ in the fight against Covid-19 that some existing research has established exists only in official Covid-19 mortality statistics. It does not show in excess mortality and in fact we find a ‘democratic advantage’ in excess mortality. Others before us have questioned the autocratic advantage (e.g., Annaka, 2021; Cassan & Van Steenvoort, 2021). Our contribution has been to add significant new evidence that is based on a different research design.

The performance of a few autocracies that, like China, managed to get through the pandemic with very low incidence and mortality rates can thus not be generalized, just as the low mortality achievement in New Zealand and Australia cannot be generalized to all democracies either, of course. Some autocracies implemented and enforced successful containment strategies but it does not follow that these countries performed well *because* they lack democratic control and civil liberties. As Stasavage (2020) points out based on historical evidence, autocracies and democracies both can be expected to have specific strengths and weaknesses when it comes to fighting emergency threats.

Our analyses provide some evidence for data fudging but it cannot prove that any specific government has *intentionally* underreported Covid-19 data. The correlation between the mortality gap and autocratic government, however, is both systematic and entirely plausible. Thus, while we cannot with certainty identify individual data fudgers, we are confident that the ‘autocratic advantage’ does not exist. Ours therefore lends additional support to a growing number of studies that suggest that lack of transparency and data fudging lie at the heart of an autocratic advantage that occurs in official records, but that does not survive and indeed is reverted once we turn to excess mortality and the gap between the two.

There is one exception to our note of caution. Belarus has clearly fabricated its Covid-19 mortality data and no sophisticated technique is necessary to detect this. Its government appears to care very little whether it is being caught in action and perhaps wishes to let the world know that they fabricate their official Covid-19 mortality records. For other countries, which employ either restrictive definitions of what constitutes a Covid-19 fatality or sophisticated algorithms to bias their Covid-19 mortality data downwards, our method of analyzing the gap between excess mortality and official Covid-19 mortality offers great promise in identifying systematic underreporting.

## Financial disclosures

None to disclose.

## Author statement

Both authors have equally contributed to all aspects of the manuscript.

## Declaration of competing interest

The authors declare that they have no known competing financial interests or personal relationships that could have appeared to influence the work reported in this paper.

## Figures and Tables

**Table:** 

	source	relevant time period	mean	s.d.	min	max
C-19 mortality per 100k people (smoothed)	https://ourworldindata.org/	monthly	6.76	10.58	0	72.27
excess mortality per 100k people	www.who.int/and for pop. data https://ourworldindata.org/	monthly	11.00	19.98	−43.74	153.65
gap excess mortality to Covid-19 mortality rate	own calculation	monthly	4.24	14.40	−44.10	139.15
incidence rate, per million people (smoothed)	https://ourworldindata.org/	monthly average	4305.09	6656.22	0	58959.51
median age	https://ourworldindata.org/	2020 and 2021	36.75	7.15	19.4	48.20
% population fully vaccinated	https://ourworldindata.org/	monthly average	12.88	22.43	0	84.05
per capita income (ln)	https://ourworldindata.org/	2020 and 2021	9.97	0.79	7.27	11.67
stringency index (t-1)	https://www.bsg.ox.ac.uk/	monthly average	56.46	20.38	0	100
government effectiveness	https://www.v-dem.net	2020 (latest available)	0.53	0.87	−1.33	2.34
liberal democracy index	https://www.v-dem.net	2020 and 2021	0.55	0.25	0.04	0.88

Note: Missing GDP per capita data for Cuba taken from https://data.worldbank.org/.

## Data Availability

Data will be made available upon acceptance for publication.

## Appendix 1

### Countries in restricted sample (based on reported total mortality data)

Albania, Algeria, Argentina, Australia, Austria, Azerbaijan, Barbados, Belarus, Belgium, Bolivia, Bosnia and Herzegovina, Brazil, Bulgaria, Canada, Chile, Colombia, Costa Rica, Croatia, Cuba, Cyprus, Czech Republic, Denmark, Dominican Republic, Ecuador, Egypt, El Salvador, Estonia, Finland, France, Georgia, Germany, Greece, Guatemala, Hungary, Iceland, India, Iran, Iraq, Ireland, Israel, Italy, Jamaica, Japan, Jordan, Kazakhstan, Kenya, Kuwait, Kyrgyzstan, Latvia, Lebanon, Lithuania, Luxembourg, Malaysia, Malta, Mauritius, Mexico, Moldova, Mongolia, Netherlands, New Zealand, Nicaragua, Norway, Oman, Panama, Paraguay, Peru, Philippines, Poland, Portugal, Qatar, Romania, Russia, Serbia, Seychelles, Singapore, Slovakia, Slovenia, South Africa, South Korea, Spain, Sri Lanka, Suriname, Sweden, Switzerland, Tajikistan, Thailand, Togo, Tunisia, Ukraine, United Kingdom, United States, Uruguay, Uzbekistan.

### Additional countries in full sample (based on either reported or predicted total mortality data)

Afghanistan, Angola, Bahrain, Bangladesh, Benin, Bhutan, Botswana, Burkina Faso, Burundi, Cambodia, Cameroon, Cape Verde, Central African Republic, Chad, China, Congo, Côte d'Ivoire, Democratic Republic of Congo, Djibouti, Eritrea, Ethiopia, Fiji, Gabon, Gambia, Ghana, Guinea, Guyana, Haiti, Honduras, Indonesia, Iraq, Laos, Lesotho, Liberia, Libya, Madagascar, Malawi, Mali, Mauritania, Morocco, Mozambique, Myanmar, Namibia, Nepal, Niger, Nigeria, Pakistan, Papua New Guinea, Rwanda, Saudi Arabia, Senegal, Sierra Leone, South Sudan, Sudan, Swaziland, Tanzania, Trinidad and Tobago, Turkey, Turkmenistan, Uganda, United Arab Emirates, Venezuela, Vietnam, Yemen, Zambia, Zimbabwe.

## Footnotes

1 hub.jhu.edu/2020/09/01/comorbidities-and-coronavirus-deaths-cdc/.

2 ourworldindata.org/excess-mortality-covid.

3 According to estimates, influenza mortality declined by 99.3 percent in the USA (IMHE, 2021).

4 https://www.who.int/data/stories/global-excess-deaths-associated-with-covid-19-january-2020-december-2021.

5 www.v-dem.net.

6 https://freedomhouse.org/and https://eiu.com.

7 https://www.systemicpeace.org/polityproject.html.
